# Edge-Based Efficient Search over Encrypted Data Mobile Cloud Storage

**DOI:** 10.3390/s18041189

**Published:** 2018-04-13

**Authors:** Yeting Guo, Fang Liu, Zhiping Cai, Nong Xiao, Ziming Zhao

**Affiliations:** 1 College of Computer, National University of Defense Technology, Changsha 410073, China; guoyeting13@nudt.edu.cn (Y.G.); zpcai@nudt.edu.cn (Z.C.); nongxiao@nudt.edu.cn (N.X.); zhaoziming93@gmail.com (Z.Z.); 2 School of Data and Computer Science, Sun Yat-Sen University, Guangzhou 510006, China

**Keywords:** edge computing, encrypted search, energy efficiency

## Abstract

Smart sensor-equipped mobile devices sense, collect, and process data generated by the edge network to achieve intelligent control, but such mobile devices usually have limited storage and computing resources. Mobile cloud storage provides a promising solution owing to its rich storage resources, great accessibility, and low cost. But it also brings a risk of information leakage. The encryption of sensitive data is the basic step to resist the risk. However, deploying a high complexity encryption and decryption algorithm on mobile devices will greatly increase the burden of terminal operation and the difficulty to implement the necessary privacy protection algorithm. In this paper, we propose ENSURE (EfficieNt and SecURE), an efficient and secure encrypted search architecture over mobile cloud storage. ENSURE is inspired by edge computing. It allows mobile devices to offload the computation intensive task onto the edge server to achieve a high efficiency. Besides, to protect data security, it reduces the information acquisition of untrusted cloud by hiding the relevance between query keyword and search results from the cloud. Experiments on a real data set show that ENSURE reduces the computation time by 15% to 49% and saves the energy consumption by 38% to 69% per query.

## 1. Introduction

In the era of the Internet-of-things, the explosive growth of smart sensor-equipped mobile devices enables the user to sense, collect, and process more data for intelligent control [1]. The sensor data increases rapidly and touches all aspects in human life. How to store the sensor data efficiently and securely is essential to make use of them to achieve intelligent control [2,3].

Mobile cloud storage refers to the access on mobile devices to cloud storage [4,5]. It enhances mobile experience that was previously impossible on resource-constrained mobile devices. Data owners are motivated to store their sensitive sensor data in cloud storage due to its great accessibility and low cost. However, mobile cloud storage brings not only convenience, but serious network security problems [6,7,8]. For example, in the environment of a smart grid, some sensor nodes (e.g., smart meters, power grid health monitoring sensors) are deployed in every family to sense and collect user fine-grained energy consumption data and send the data to the cloud storage which the power grid operators run [9]. It enables the power grid operators to effectively manage the demand and supply of electricity, which is significant in withstanding large-scale blackouts caused by insufficient power supply during peak times. However, it also benefits attackers to know about the resident power consumption mode, and increases the possibility of a crime, like burglary. Thus, it is necessary to encrypt the sensor data before being outsourced to the third party, and also to ensure that authenticated users can search encrypted data.

Although traditional encrypted search schemes (e.g., [10,11,12,13], to list a few) provide a keyword search without decryption, they are more suitable for the users with personal computers, because in these schemes, the client needs to process some computation-intensive tasks in order to reduce sensitive information leakage and improve the data security. When it comes to mobile devices, the mobile client cannot afford the considerable computing workload and communication overhead caused by these tasks for resource constraints. Some researches [14,15,16] propose to offload these tasks which should be processed by clients onto the cloud in order to obtain a higher efficiency. However, this method divulges the relevant degree between keywords and documents to the untrusted cloud, which may lead to potential threats to users’ data security [17,18,19]. Consequently, there are conflicts between the performance and the users’ data security when using the mobile device to access the encrypted search service.

With the fast-growing development of edge computing [20], many novel methods appear to solve the resource-constrained problem in mobile applications. “Edge” refers to computing, storage, and network resources available during the data transmission from the data source to the cloud [21]. They could be tablets, laptops, home PCs, or even Wi-Fi routers [22]. Developers can select appropriate local resources as the edge server to release the mobile device from heavy computation tasks and migrate partial cloud services to reduce latency and protect privacy. The edge server typically has more computation resources and energy than most of the sensor-equipped mobile devices and is more easily accessible than the remote cloud. Besides, if the edge server provides the interface for multiple types of sensors, it collects data from them and processes data more efficiently, which is of great significance in scenarios with multiple types of sensors and promotes the large-scale application of sensors. More importantly, users could deploy some available, controllable, trustworthy, and relative resource-rich local devices nearby as the edge server to execute necessary privacy protection algorithms which are not suitable to run on the cloud for security considerations. Consequently, edge computing may be another way to apply encrypted search schemes over mobile cloud storage. Therefore, our research focuses on taking advantage of the edge server to reduce the conflicts as we mentioned in the last paragraph and enhances both the efficiency and security of encrypted search.

In this paper, we introduce a new architecture, ENSURE, for mobile cloud storage applications. Inspired by the characteristic of edge computing, ENSURE offloads the computation-intensive tasks to appropriate nearby devices to increase the performance efficiency. Moreover, benefiting from the trustworthiness of nearby devices, ENSURE allows the edge server to replace the cloud to handle the file index search process, which reduces the information acquisition of the untrusted cloud. ENSURE achieves the flexible use of edge computing resources, which is embodied in that the edge resources are a plug and play component in ENSURE, that is, they are optional in our search scheme. In the last step, ENSURE adopts a ranked keyword search algorithm as its basic encrypted search scheme to respond to the client’s request with the top-k relevant files. For example, Bob stores a set of documents on an untrusted cloud using a searchable encryption technique. One day, he uses ENSURE at home to search the top-10 relevant files related to the keyword “security” by his smartphone. ENSURE could leverage the trusted local devices at home (e.g., home PC) to help his smartphone to complete the search process efficiently and securely. But, if he uses ENSURE in the scenario where he cannot access any trusted local device, ENSURE could also complete the search process with the traditional encrypted search scheme, which may increase the search time and energy consumption.

This paper proposes ENSURE, an efficient encrypted search architecture which offloads computing intensive tasks onto edge servers to minimize the file search/retrieval time and the energy consumption. Our experimental results show that ENSURE reduces the file search/retrieval time by 15–49% and the energy consumption by 38–69% in comparison with the traditional encrypted search scheme. Moreover, ENSURE uses the edge server to search the file index, but retrieves documents from the cloud. So, the cloud could learn nothing about the relevance between the query keyword and the search results, which reduces the information acquisition of the cloud and prevents an untrusted and uncontrollable cloud from eavesdropping on the user’s data.

The paper is organized as follows: Section 2 introduces the related works. Section 3 presents the traditional encrypted search architecture over cloud data. Section 4 describes the design of ENSURE. The evaluation results are given in Section 5. Finally, we summarize our conclusion in Section 6.

## 2. Related Work

Mobile cloud storage faces various security threats. Firstly, the cloud provider may be untrusted and want to profit from user information [23,24]. Secondly, several users share the same physical infrastructure in cloud storage, thus malicious users can obtain other user information through attacks such as unauthorized access, reverse control, and memory leaks [25]. Lots of privacy protection algorithms are proposed to resist the security risks caused by the untrusted cloud.

Encrypted search schemes are one of the privacy protection algorithms. They can be categorized into two main classes: boolean keyword search and ranked keyword search. The former selects files only based on whether the keyword appears in the content of the file and is not concerned about any relevance of the files in the search result (e.g., [12,13,14,26]). The latter records the relevance scores to compare the relevance of files to the searched keyword and then replies with the top-k relevant files as a response (e.g., [11,15,16]). It greatly improves the efficiency of extracting useful information and accuracy. Therefore, it has been widely used by cloud storage and has attracted many researchers to develop it.

Most of the latest studies about ranked keyword search focus on the cloud storage scenario. In [11], Swaminathan et al. developed a framework for a confidentially-preserving rank-ordered search. In this scheme, the computation task of the relevance scores is assigned to the client side, which increases the client’s workload as a sacrifice to ensure the data security. In [10], Zerr et al. proposed the concept of r-confidentiality as the degree of information leaked from an index, and proposed a system that allows for tunable index confidentiality and efficiency. But it only allows the client to decrypt the posting list and perform a top-k relevant search. This kind of research all assigns a heavy workload to the client, so it is not suitable for mobile cloud storage which uses resource-constrained mobile devices to access the cloud service.

Considering the resource constraints in a mobile device, Miettinen and Nurminen in [27] pointed out that offloading some computing intensive tasks onto a cloud could be an effective way of dealing with this issue. Wang et al. in [26] presented a secure ranked keyword search over encrypted cloud data which used one-to-many mapping Order Preserving Encryption to encrypt the index of the file set. This design allows an efficient server-side ranking which reduces the client-side workload. Li et al. in [14] used cloud computing to improve the encrypted data search performance. Bowers et al. [28] proposed a distributed cryptographic system where the safety and retrieval of stored files are proven by a set of cloud servers. Wang et al. [15] introduced a secure ranked keyword search over encrypted cloud data. However, information leakage relating to the relevance between keywords and documents exists in these schemes, which could result in untrusted cloud providers obtaining the major term of stored files.

In the era of the Internet of Things, edge computing is rapidly emerging because edge servers build a bridge between resource-constrained mobile devices and the cloud. Since applications can access infrastructure and application services provided on-premises [29], and edge servers and mobile devices are usually in the same LAN, edge severs would be conductive for mobile devices to implement the necessary privacy protection algorithm and protocols efficiently with their relatively adequate resources when data owners want to outsource massive local data from mobile devices to the cloud. Wadood Abdul et al. [30] proposed implementing visual cryptography and zero-watermarking algorithms on edge servers to encrypt the face images that are to be uploaded to the cloud. This prevents the untrusted cloud from obtaining and abusing user biometrix content, and also ensures the image quality, which means that the result of face recognition is not affected by image encryption. Maher Jridi et al. [31] also offloaded the image compression and encryption tasks to the digital gateway on the edge of network. Manisha Jindal et al. [32] used a trustworthy edge server to implement a secure forward encryption algorithm to prevent data from being acquired by unauthenticated users and untrusted service providers. Therefore, edge servers can be a key component in the secure data processing framework for mobile cloud storage and resemble the cloud trusted domain which is introduced in [33].

Therefore, we regard the ranked keyword search scheme as a basis to establish an efficient and secure encrypted search architecture over mobile cloud storage with the help of edge computing.

## 3. Encrypted Search in Cloud Storage

### 3.1. Traditional Encrypted Search over Cloud Data

**Figure 1:**
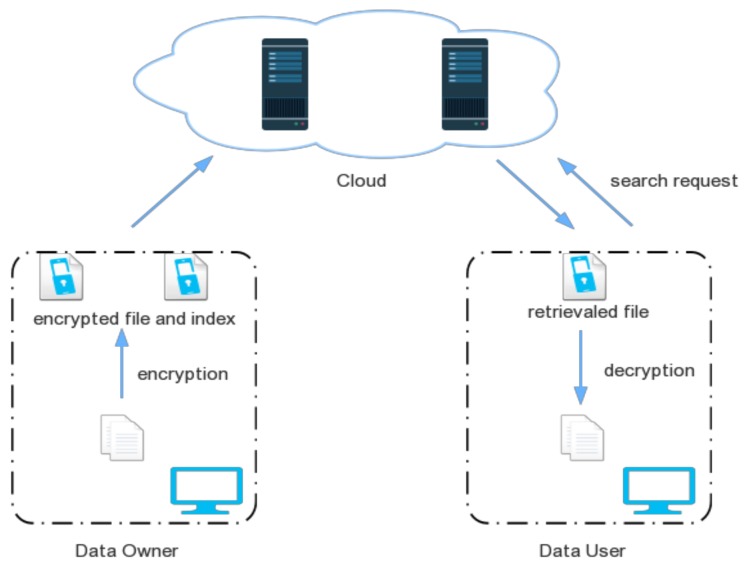
Traditional Encrypted Search Architecture.

The traditional encrypted search architecture over cloud data is illustrated in Figure 1. Data owners and data users have different procedures to be completed. Index generation and file encryption should be processed by data owners, while data users should accomplish encrypted file search and retrieval.

#### 3.1.1. Index Generation and File Encryption

When the data user intends to perform a keyword search on stored files, downloading all these files from the cloud and decrypting them to find out the top-k relevant files on the client side wastes great time and energy. The solution can be storing an extra file index in the cloud for searching. The highly effective index mechanism is the base of the highly effective search. So, the data owner first extracts distinct keywords from stored files and then builds a secure file index, which is usually an inverted file [34] consisting of a sequence of posting lists. Specifically, every keyword needs to be encrypted and hashed to fix its entry in the index, and the related posting list also needs to be encrypted against unauthorized access. The file encryption procedure is simple for mobile devices, just encrypted in a way that is consulted with the data user in advance. Lastly, both the file index and the encrypted files are sent to the cloud.

#### 3.1.2. Encrypted File Search and Retrieval

**Figure 2:**
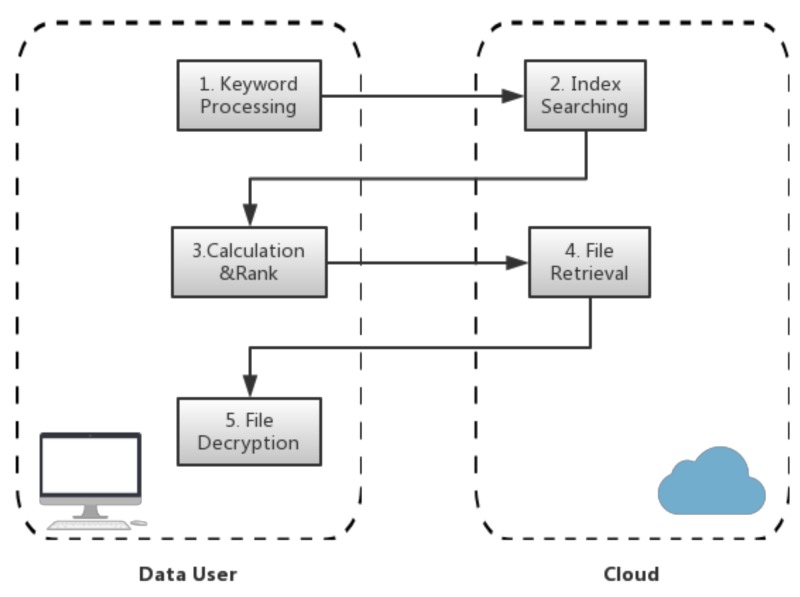
Process of Encrypted File Search and Retrieval.

In the process of search and retrieval, the cloud helps the user to find the top-k relevant files according to the keyword which the user submits. It is noteworthy that only authorized users who are capable of generating the trapdoor are entitled to search the encrypted files. The trapdoor is used to search for the intended keyword in the cloud. The process is divided into five steps. For better understanding this process, we illustrate the five steps in Figure 2, and present the related computational components for these steps.

1. Keywords Processing: When users submit the keyword, the client first processes the keyword to generate the trapdoor of the keyword. Then, the client sends a search request (the trapdoor of the keyword) to the cloud server.
2. Index Searching: On receiving the search request, the cloud uses the trapdoor to gain entry to the file index. Then, the posting list related to the keyword is sent back to the data user.
3. Calculation&Rank: The data user decrypts the posting list and calculates the relevance scores to find the top-k relevant files, and then sends a request to retrieve the files.
4. File Retrieval: The cloud server finds the target files and sends them back to the data user.
5. File Decryption: The data user decrypts the target files to recover the original data.

### 3.2. Challenges in Mobile Cloud Storage

Efficiency Challenges: in the traditional encrypted search scheme, data processing tasks are mainly handled by the user’s device, and many of the tasks are computation-intensive, such as decryption and hash. However, the encrypted file search and retrieval procedure is not suitable for mobile devices for two reasons. It is a heavy burden to decrypt the index and calculate the relevance scores for a mobile device. The mobile device may take more time to complete the calculation step than a powerful PC, so the query response time increases significantly and user experience is degraded. From the aspects of energy consumption, this design is also inappropriate. More latency and energy consumption will be introduced by a large amount of communication between the mobile device and cloud, and at the same time, the large traffic consumption may cost a payable traffic fee. Therefore, if we directly transplant this traditional solution into mobile cloud storage, that may lead to a low-efficiency system and a poor user experience for mobile users.

Security Challenges: offloading some computation intensive tasks onto the cloud is a popular way to compensate for the drawback of the resource-constrained mobile devices and is widely used in many mobile applications [35]. But in the scenario of an encrypted search, we usually assume that the cloud is “honest-but-curious” [12], and attempts to access the underlying plaintext of users’ data. Additionally, there are many attack models [17,36,37] to use the information leakage against the encrypted search scheme. A desired security scheme should minimize the information leakage. Therefore, offloading computation onto the cloud is not an appropriate way to improve the efficiency of the encrypted search scheme in consideration of the data security. Moreover, in an encrypted search scheme, the computation in the untrusted cloud should be as little as possible in order to minimize the sensitive information leakage. For example, in the traditional ranked keyword search scheme, if the cloud server is in charge of the calculation task of the relevance scores, a more practical performance may be achieved. However, the cloud may deduce the association between keywords and encrypted files, which may cause the major subject of a document learned by the cloud [17]. In that case, a great threat to user’s data security may be posed.

We have made clear the challenges ahead, so we design ENSURE to achieve the following goals, and the detailed system design will be introduced in the next section.

1. Improve the performance efficiency of traditional encrypted search method, which includes reducing the file search/retrieval time and energy consumption.
2. Try our best to minimize the information acquisition of the curious cloud.

## 4. ENSURE System Design

To effectively address the challenges and achieve our goals, we introduce a new architecture named ENSURE. In this section, we first discuss why we have chosen edge computing to solve the problems, and then introduce the design idea of our system and the process of file search and retrieval in ENSURE. Finally, we discuss the performance efficiency of ENSURE.

### 4.1. Edge Computing

Edge computing refers to the enabling technology which allows computation and service to be hosted at the edge of the network, and plays the role of the middle layer between the data source and cloud. There are various types of resources at the edge of the network. Typically, the mobile terminal whose energy and compute capability are limited (e.g., sensors, mobile phones, and tablets) is regarded as an edge node, while the device which has a constant energy supply and continuous network connectivity (e.g., home PCs, laptops) is regarded as an edge server. Note that edge node and edge server are relative concepts. There is no clear boundary between the two.

It brings new opportunities to the scenario with multiple types of sensors. This is because the edge sever could pull some tasks up from sensor-equipped mobile devices and some appropriate services can also be pushed from the cloud toward the edge. For example, in the smart home system, lots of wireless sensor-equipped mobile devices are deployed to sense, collect, and process data to achieve intelligent control, such as the temperature sensor and pyroelectric infrared sensor. If the data is leaked, the attacker could judge whether the owner is at home, or get other useful information. But such mobile devices are constrained in computation and storage resources, resulting in the lack of data protection. There are numerous similar scenes where multiple sensor-equipped mobile devices move in a certain region, such as hospitals and supermarkets. Edge computing suggests users choose one of the available, controllable, trustworthy, and relative resource-rich local devices to be the edge server to settle sensor-equipped mobile devices’ annoyance. Additionally, the edge server would provide the interface for various sensors to gather and process sensed data more efficiently. With this approach, sensor-equipped mobile devices improve the performance and prolong the time of use at the expense of the energy of the edge server. Edge servers usually have more energy reserve and are easier to provide with an energy supply, and mobile devices with less energy reserve need to be recharged frequently. What is worse, users may have to recharge several powerless mobile devices simultaneously, which really reduces the user experience. Meanwhile, the extra overhead is generated by the interaction between edge servers and mobile devices, but it is quite small relative to the benefit that the edge server brings.

### 4.2. The Basic Idea of ENSURE

Inspired by the characteristic of edge computing, ENSURE leverages edge servers to achieve an efficient and secure ranked keyword encrypted search. It is based on the following assumptions:

1. Sensor-equipped mobile devices move in some certain region.
2. One of the available, controllable, trustworthy, and relative resource-rich local devices in that region plays the role of edge server.
3. The data transmission between mobile devices and the edge server is safe.

The basic idea of ENSURE consists of two parts. The first part aims to improve the efficiency of the encrypted search. We propose to offload the workload of Calculation&Rank and File Decryption (steps 3 and 5 in the process of the search and retrieval introduced in Section 3) onto the edge server, because they are both computation-intensive tasks. The edge server could deal with these tasks in less time because of the superiority in resources. Mobile devices are released from heavy computation tasks, which decreases the time and energy consumption in the encrypted search. What is more, they could make better use of the energy to sensor data.

In the second part, we focus on data security protection from the untrusted cloud. Our idea is to divide the encrypted data search and retrieval into two parts which have different computational components. More specifically, we assign the edge server to process the encrypted data search, and the data retrieval is processed by the cloud without changes. Therefore, the untrusted cloud cannot get any information about the keywords the user submits and the relevance between the query keyword and search results. The detailed design of how ENSURE addresses the efficiency and secure challenges will be introduced in the next subsection.

### 4.3. Process of File Search and Retrieval in ENSURE

To offload the encrypted data search to the edge server, the edge server should download the encrypted file index which is stored in the cloud and synchronize it with the cloud. This is entirely feasible for three reasons. Firstly, the edge server has a relatively sufficient storage space to store the file index, which is much smaller than files. Secondly, the edge server usually has a stable network condition for file index synchronization. Last but not least, the update of data is far less frequent than data search, so one synchronous interaction between the edge server and cloud may support multiple queries on average.

**Figure 3:**
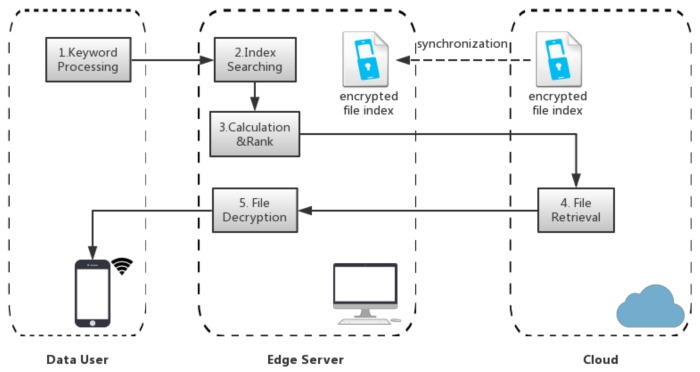
ENSURE’s Process of File Search and Retrieval.

After the edge server downloads the file index, the workload of Index Searching (step 2 in the process of file search and retrieval introduced in Section 3) can be handled by the edge server. So, the search request is sent to the edge server but not the cloud. The data user could send the key to the edge server through a secure connection, and the key is used to decrypt the posting list related to the query keyword. The process of encrypted file search and retrieval in ENSURE is shown in Figure 3 and follows the steps:

1. Keyword Processing: Since the data owner permits the data user to access the data, the data user could encrypt and hash the keyword to generate the search request (a trapdoor of the keyword) in their mobile device when he wants to search the top-k relevant files involving the keyword. Following this, the mobile device would send the request to the edge server and wait for the response from the edge server.
2. Index Searching: On receiving the request, the edge server first synchronizes the encrypted file index from the cloud in case of the file index update. Then, it would decrypt the file index and search the matching posting list in the index based on the search request. The posting list contains the information (e.g., word frequency) of files that involve the keyword.
3. Calculation&Rank: The edge server obtains and decrypts the posting list corresponding to the keyword, then uses the information in the posting list to calculate the relevance scores to find the top-k relevant files. Lastly, since all the files are still stored in the cloud storage, a request should be sent to the cloud server in order to retrieve these files.
4. File Retrieval: The cloud finds the retrieved files and sends them back to the edge server upon request.
5. File Decryption: The edge server receives these files from the cloud. After decryption, the top-k relevant files are sent to the mobile device.

Compared with the traditional scheme, we conclude that offloading the relevance score calculation and file decryption onto the edge server releases the user’s mobile device from a heavy computation load, which is the main bottleneck to reducing the file search/retrieval time and saving energy. Moreover, the edge sever blocks the access of the curious cloud to the relevance between the query keyword and the search results because the index searching process is handled by the edge server and the curious cloud only retrieves target files upon request, but has no way to learn the keywords corresponding to these files.

Note that the edge server is a plug and play component in ENSURE, so if users cannot access the edge server, ENSURE can also support the encrypted search service with the traditional method introduced in Section 3.

### 4.4. Performance Efficiency of ENSURE

As our goals described, we intend to propose a solution for an encrypted search over the mobile cloud with the minimal file search/retrieval consumption in mobile devices. So, in this subsection, we theoretically discuss the improvement in time and energy consumption compared with the traditional method.

The process of file search and retrieval in two schemes is composed of five steps. These steps are listed below.

1. Keyword Processing: In two schemes, this step makes no difference, so the execution time is equal.
2. Index Searching: The index search time of ENSURE is faster than the traditional method because in the traditional method, the client has a round-trip communication with the cloud to send the processed keyword and retrieve the posting list corresponding to the query keyword, but ENSURE only needs the client to send the search request to the edge server for the local index and wait for the results.
3. Calculation&Rank: The computation workload in this step is an increasing function of the document frequency (the number of files containing the keyword). In the traditional method, the high document frequency results in the rapid growth of the execution time of Calculation&Rank because the resource-constrained mobile device cannot afford this heavy workload. As for ENSURE, it allows the edge server to implement this step. With the relative abundant computing resources, the edge server is slightly effected by the high document frequency and keeps the growth of execution time relatively slow.
4. File Retrieval: The time consumption of this step mainly depends on the network bandwidth accessing the cloud. The higher the bandwidth, the faster the speed of file retrieval. In both of the schemes, the cloud sends the relevant files back upon request. The files are only determined by the keyword. Thus, if the network condition is the same, the time consumption exhibits almost no differences between the two schemes in this step.
5. File Decryption: In ENSURE, the file decryption is handled by the edge server, so the file decryption time of ENSURE is smaller than the traditional method which leverages the mobile device to decrypt the files. However, after decrypting the files, the edge server should send the original data back to the mobile device, which is not needed in the traditional method. The extra transmission latency is too small to disturb the efficiency improvement since the edge server is in the same LAN with mobile devices and the distance between them is quite short.

As for the performance of energy consumption, the client in ENSURE only needs to handle the Keyword Processing and afford some communication overhead such as establishing a secure connection to transmit the secret key. Thus, the superiority of ENSURE is easily observed.

### 4.5. Security of ENSURE

Security is also the main characteristic of our proposed design. In the subsection, we analyze the security of ENSURE compared to the traditional method in detail from the perspective of the information interaction between the cloud server and the client.

In the traditional method, two information interactions exist between the cloud server and the client, which happens in Index Searching and File Retrieval, respectively. In the process of Index Searching, the cloud server gets the keyword from the client and replies with the corresponding post list to the client. In the process of File Retrieval, the cloud server receives the request of target files and returns these files. Based on the keyword and target files, the untrusted cloud is most likely to obtain the major term of files by deducing the association between them.

In ENSURE, there is no direct information interaction between the cloud server and the client, because the edge server acts as a middleman, which blocks the cloud server from getting keywords from the client. Thus, the only information the cloud server obtains in the process is the list of top-k relevant files. Since the cloud server could not establish the connection between keywords and relevant files, the major term of files would not be leaked. Therefore, ENSURE resists the security risk from the uncontrollable and untrusted cloud.

## 5. Evaluation

In this section, after describing the experimental environment in Section 5.1, we discuss the evaluation of ENSURE performance on the file search/retrieval time (FSRT) and energy consumption in Section 5.2 and Section 5.3. For comparative purposes, we also implement the traditional method in each experiment.

### 5.1. Experimental Environment

To evaluate the ENSURE system, we choose 5402 Request for Comments (RFC) documents with the type of TXT to be our data set. These RFC documents are collected from The Internet Engineering Task Force (IETF) [38]. The experimental testbed is comprised of a VM with a Quad vCPU; a PC with an Intel Core i5-4590 CPU (3.3 GHz, Golden Field Industrial Co., Ltd., Dongguan, China), 8 G memory, and a network with a 10 Mbps rate; and an android smartphone with a Snapdragon 810 CPU, 3 G RAM, and 10 Mbps TD-LTE network (Xiaomi Inc., Beijing, China). They play the role of the cloud server, the edge server, and the mobile device, respectively. Additionally, the smartphone installs an android application which uses the ENSURE system to search and retrieve the relevant files. As for the connection among them, the mobile device accesses the edge server via a 50 Mbps WLAN, and the edge server connects to the cloud via a high-speed 5 GHz 802.11n network. Besides the Trepn Power Profiler [39], a power profiling tool for the smartphone with a Qualcomm CPU is installed on the smartphone to observe the energy consumption. Furthermore, in terms of the implementation details of the ranked keyword search scheme, the algorithm theory in [8] is referred to as the traditional method. ENSURE adjusts the algorithm execution units based on the system architecture as mentioned in Section 4.3.

### 5.2. File Search and Retrieval Time

In the experiment, we want to search the top-three relevant documents for the selected keywords and measure the FSRT. To analyze the impact of the document frequency on the FSRT simultaneously, we select five keywords with different document frequencies ranging from 97 to 2137. The selected keywords and their related document frequency are shown in Table 1.

**Figure 4:**
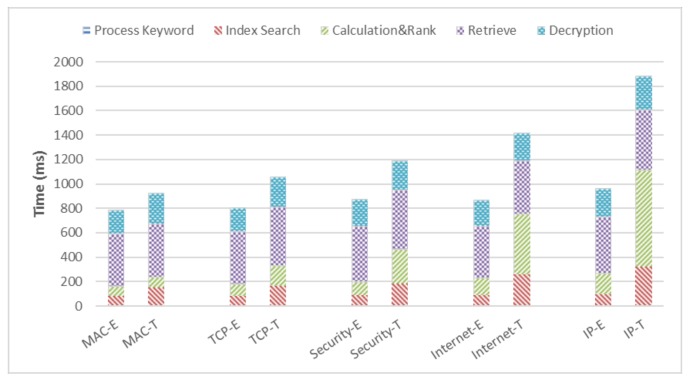
FSRT of ENSURE and Traditional Method: E represents ENSURE, T represents traditional method.

The smartphone uses the application introduced previously to submit the search request of each keyword to the edge server respectively. Then, the smartphone waits for the edge server to handle the request and sends the top-three relevant files back. The process could be divided into five parts, which are process keyword, index search, calculation&rank, retrieve, and decryption, as we described in Section 4. Thus, the FSRT is equal to the sum of the time that each part takes. We measure the FSRT of each keyword in two schemes, and the experimental results are shown in Figure 4. The results show that ENSURE could saves about 15% of the FSRT for the low document frequency keyword and 49% for the high document frequency keyword. With the frequency increasing, the difference in performances among the two schemes are widened. More specially, it is the time to complete the index search, calculation&rank, and decryption steps that results in the widened difference. The three parts are all computation-intensive tasks and would increase the burdens of the smartphone when searching high document frequency words. Offloading these tasks onto the edge server which is equipped with relatively abundant computing resources benefits from reducing the FSRT. As for the time to process the keyword, the smartphone generates the trapdoor of the keyword according to the security protocol. While it is relatively easy to implement and irrelevant to the document frequency, the time to this part remains short and stable. Additionally, the time to retrieve is determined by the network bandwidth and the size of the relevant files. Since the smartphone requests the top-three relevant files and there is little difference in the size of the files, the time to retrieve is almost unchanged.

### 5.3. Energy Consumption

**Figure 5:**
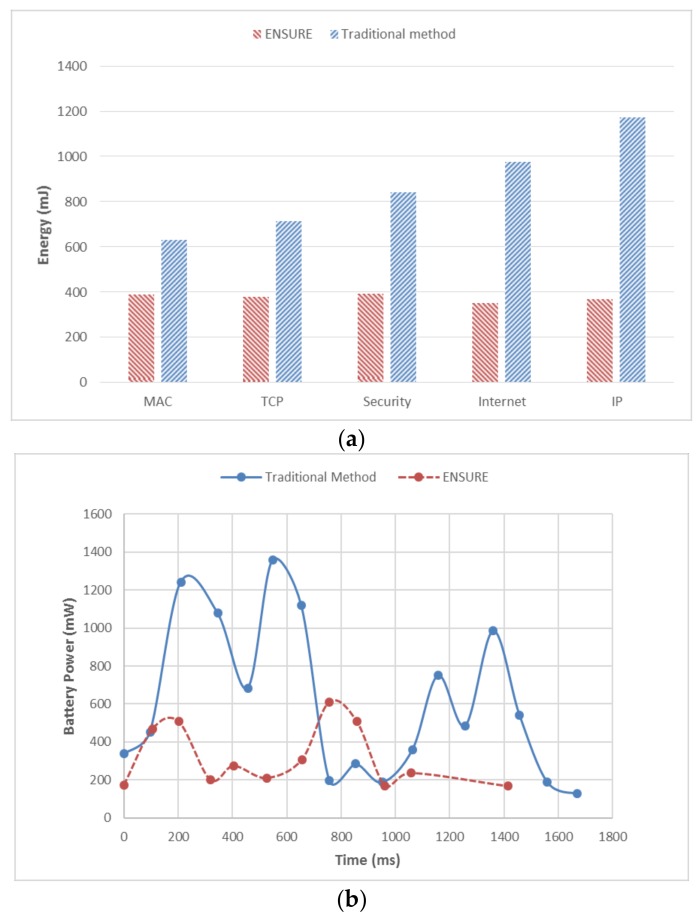
Performance of Energy Consumption. (**a**) Energy consumption of ENSURE and Traditional Method; (**b**) power versus time while searching keyword “Internet”.

As described above, we focus on the energy consumption on the smartphone in this subsection to prove that ENSURE is more energy-saving than the traditional method. Five sets of experiments are conducted to measure the energy consumption of two schemes with different document frequency words. The results are shown in Figure 5a. From the smartphone’s perspective, ENSURE enables the smartphone to reduce the energy consumption by 38–69%. The energy consumption of ENSURE fluctuates slightly with the change of document frequency, while the energy consumption of traditional methods increases sharply.

For further analyzing the energy consumption in detail, we evaluate the power versus time. The power-time diagram is illustrated in Figure 5b, and the data is collected in the case of using the keyword “Internet” to search the top-three relevant documents. As illustrated in Figure 5b, there exists four major energy consumption points in the traditional method and two in ENSURE. Through combined analysis of the FSRT in Section 5.2, we could easily find that the four points correspond to Index Searching, Calculation&Rank, File Retrieval, and File Decryption, respectively. Furthermore, they require high demands on computation resources and consume most of the energy. Since Index Searching, Calculation&Rank, and File Decryption are offloaded onto the edge server in ENSURE, the smartphone is released from heavy computation. The two major energy consumption points in ENSURE are the communication overhead with the edge server and File Retrieval; but both of them would not disturb the smartphone too much. Overall, the energy consumption of ENSURE is significantly reduced and less affected by document frequency of the keyword.

## 6. Conclusions

This paper presents ENSURE, a new architecture for supporting an efficient and secure encrypted search over mobile cloud storage. In particular, ENSURE makes use of powerful edge computing resources to handle computation-intensive tasks and deal with sensitive data. In that way, ENSURE could achieve a high-efficiency performance and minimize the information acquisition of the curious cloud. To that aim, this paper has shown how we carefully redesign the search procedure and evaluate it with the comparison of the traditional method. Our experimental results show that ENSURE could result in much faster queries and significant energy saving, especially when the document frequency of the searching keyword is high in the data set.

However, our current work still results in some possible extensions. In this paper, we take advantage of the fully trusted edge resources such as our own laptop or home PCs, but not all edge resources are fully trusted. So, it would be of significance to find a secure way to leverage these edge resources in the future.

## Figures and Tables

**Table 1:** The Document Frequency of Each Keyword.

Keyword	Document Frequency
MAC	97
TCP	277
Security	586
Internet	1291
IP	2137
